# Structure, Expression, and Function of ICAM-5

**DOI:** 10.1155/2012/368938

**Published:** 2012-01-23

**Authors:** Heping Yang

**Affiliations:** ^1^Department of Neurology, The No.5 People's Hospital of Shangrao, Jiangxi 334000, China; ^2^Ruikang Hospital, Guangxi Traditional Chinese Medical Collage, Guangxi 530011, China

## Abstract

Cell adhesion is of utmost importance in normal development and cellular functions. ICAM-5 (intercellular adhesion molecule-5, telencephalin, TLN) is a member of the ICAM family of adhesion proteins. As a novel cell adhesion molecule, ICAM-5 shares many structural similarities with the other members of IgSF, especially the ICAM subgroup; however, ICAM-5 has several unique properties compared to the other ICAMs. With its nine extracellular Ig domains, ICAM-5 is the largest member of ICAM subgroup identified so far. Therefore, it is much more complex than the other ICAMs. The expression of ICAM-5 is confined to the telencephalic neurons of the central nervous system whereas all the other ICAM members are expressed mostly by cells in the immune and blood systems. The developmental appearance of ICAM-5 parallels the time of dendritic elongation and branching, and synapse formation in the telencephalon. As a somatodendrite-specific adhesion molecule, ICAM-5 not only participates in immune-nervous system interactions, it could also participate in neuronal activity, Dendrites' targeting signals, and cognition. It would not be surprising if future investigations reveal more binding partners and other related functions of ICAM-5.

## 1. Introduction

The immunoglobulin superfamily (IgSF) comprises over 100 members are in vertebrates and most of its members expressed at the cell surface. Molecules carrying immunoglobulin (Ig) domains are endowed with broad functions which include cytoskeletal organization, endocytosis, adhesion, migration, growth control, immune recognition, viral attachment, and tumor progression. The ever growing number of newly discovered Ig molecules has made this superfamily increasingly complex.

Intercellular adhesion molecules (ICAMs) are a subset of the IgSF that bind to leukocyte *β*2 integrins. There are now five members of this family: ICAM-1 has a relatively broad distribution in different tissues, whereas ICAM-2, -3, -4, and -5 show more restricted patterns of tissue expression. The five ICAMs share many structural and functional features [1]. A 26 kb region on human 19p13.2 contains ICAM-1 (MIM 147840), ICAM-4, and ICAM-5 (MIM 601852) genes [2]. Structurally, all ICAMs have lg-like domains; functionally, most ICAMs participate in immune system interaction. Notably, ICAM-5/TLN may play important roles both in the immune and nervous systems.

In this review, the main focus is placed on the structure, expression, and function analysis of mouse, rabbit, and human ICAM-5. Here I will discuss the characterizations of ICAM-5 gene and protein structure and expression as well as function in the nervous system.

## 2. Structure of ICAM-5

ICAM-5 is a membrane glycoprotein that was first purified from the telencephalic regions of the rabbit brain, thus named ICAM-5 [3]. Since then, the cDNAs of rabbit [4], mouse [4], and human [5] ICAM-5 have been cloned. The cDNA cloning reveals that ICAM-5 belongs to the Ig superfamily, comprising an NH_2_-terminal signal peptide, a characteristic extracellular region with nine Ig-like domains, a single transmembrane region, and a COOH-terminal cytoplasmic tail [4, 5]. The overall length of cDNA is about 3.0 Kb, and consists of 11 exons in all mammal species. The amino acid sequence of different species revealed that it is a type I integral membrane glycoprotein of 130 kDa, with a 107 kDa polypeptide composed of a signal peptide (26-27 amino acids), a large extracellular region (792–805 amino acids), a transmembrane region (28 amino acids), and a relatively short cytoplasmic tail (59–64 amino acids) [4–6]. The extracellular portion contains nine tandem repeats of C2-type Ig domains, rendering a member of the IgSF.

### 2.1. Structure of the Mouse ICAM-5 Gene and Protein

Mouse ICAM-5 gene is located in the ICAM gene cluster containing ICAM-1, ICAM-4 and ICAM-5 within 30 kb on the mouse chromosome 9. The nucleotide sequence of the 5′-flanking region of mouse gene is highly homologous to that of human and dog orthologs, suggesting the presence of important regulatory elements for its transcription [6]. The mouse ICAM-5 is linked to low-density lipoprotein receptor (Ldlr), ICAM1, and amyloid precursor-like protein 2 (Aplp2). It contains approximately 3.9 kb of the 5′-flanking region, 6.3 kb of the gene, and 2.6 kb of the 3′-flanking region.

The entire protein-coding sequence was divided into 11 exons. There was a correlation between exon-intron gene structure and functional domains of ICAM-5 protein. Exon 1 included a translation initiation methionine (ATG) followed by a putative signal peptide. Exons 2–10 were clustered within 4 kb and encoded individual nine Ig-like domains of the extracellular region. Exon 11 contained both transmembrane and cytoplasmic regions of ICAM-5 followed by a polyadenylation signal (AATAAA) 116 bp downstream from the stop codon (TGA) [4].

Using total ICAM-5-RNA from mouse telencephalon, in which mRNA is specifically expressed [4, 7], strongly positive signals were detected, located at 92, 94, and 95 bp upstream from the ATG start codon, whereas no specific signal was found with total ICAM-5-RNA from mouse cerebellum. This indicates that nucleotides 92–95 bp upstream from the start codon are potential transcription initiation site of the mouse ICAM-5 gene.

Of the three potential sites, in particular nucleotides around adenine, matched a consensus sequence (YYCAYYYYY) for a strong initiator element [8]. This initiator sequence has been proposed to function as an alternate TFIID-binding site in genes lacking a TATA box [9]. Two CAAT boxes (−23 to −20) and (−36 to −33) and a binding site for CAAT/enhancer binding protein (C/EBP) (TKTGGWNA) (−47 to −40) [10] were found in the upstream vicinity of the transcription start site [4]. In mouse ICAM-5-cDNA, a potential polyadenylation signal (AATAAA) was found at nucleotides 2910–2915. Analysis of the promoter region of the mouse ICAM-5 gene revealed a consensus strong initiator element for TATA-fewer genes, two CAAT boxes, and numerous putative transcription factor binding sites. Of note are four E-box (CANNTG) and two N-box (CACNAG) sequences clustered at positions −949 to −365, four copies of Nkx-2.5 sites (CWTAATTG and TYAAGTG) [11], three copies of Sp1 sites (GRGGCRGGGW) [12], two copies of c-Ets-1 sites (CMGGAWGYN) [13], a heat shock factor-2 binding site (HSF2) (GAANNWTCK) [14], an Oct-1 site (RTAATNA) [15], a GC box (KRGGCGKRRY) [16], an interleukin 6-induced nuclear factor-binding site (NF-IL6) (AGTGANGNAA) [17], an Evi-1-binding site (ACAAGATAA) [18], and a Pbx-1-binding site (ANCAATCAW) [19]. The E- and N-boxes have been shown to be binding sites for transcription factors which belong to the basic helix-loop-helix superfamily, such as MASH-1, HES-1, -2, -3, -4, -5, MATH-1, -2, and E12/E47 [20, 21]. The proximal region of mouse chromosome 9 shares regions of homology with human chromosomes 19p and 11q [20]. The nucleotide sequence of the 5′-flanking region of mouse gene is highly homologous to that of human and dog orthologs', suggesting the presence of important regulatory elements for its transcription [6].

All 30 Cys residues (28 in the extracellular region; 2 in the cytoplasmic region) were completely preserved in mouse ICAM-5 protein [4]. The structure of ICAM-5 is most closely related to those of ICAM-1 and -3 with more than 50% amino acid identity in the distal five Ig-like domains [4, 5]. The first to fourth Ig-like domains of mouse ICAM-5 are aligned with the corresponding domains of mouse ICAM-1 [22], mouse ICAM-2 [23], and human ICAM-3/R [24, 25]. The fifth to eighth immunoglobulin-like domains of mouse ICAM-5 protein are aligned with the fifth domain of mouse ICAM-1 and human ICAM-3/R [4]. The domain I of mouse ICAM-5 shares 26%–29% amino acid identity with that of ICAMs. In particular, domains II, III, and IV of mouse ICAM-5 share 60%, 66%, and 64% amino acid identity with the corresponding domains of ICAM-3/R, respectively. Domains V, Vl, VII, and VIII in mouse ICAM-5 protein are similar to each other, with pairwise identities in the range of 31%–49%. Although the previous reports on ICAMs disagree in setting a possible junction between domains IV and V [26–28], the repeating structure in domains V–VIII indicates that domains IV and V contain 2 and 4 Cys residues, respectively. The cytoplasmic sequence of mouse ICAM-5 comprises 59 amino acids [4]. In addition, the cytoplasmic domain of ICAM-5 contains one and two potential sites for phosphorylation by protein tyrosine kinases and by casein kinase II, respectively, (Tyr-868, Ser-875, and Ser-903 in mouse).

The distal-most Ig-like domain of ICAM-5 contains 4 cysteine residues that are capable of forming two intradomain disulfide bridges. Similarly spaced cysteine residues in the NH_2_-terminal Ig-like domains are seen in other members of the Ig superfamily, such as ICAM-1 [26, 27], ICAM-2 [29], ICAM-3 [24, 25], LW (ICAM-4) [30–32], and MAdCAM-1 [33]. The NH_2_-terminal Ig-like domains of ICAM-1, -2, -3, -4, and ICAM-5 contain characteristic four cysteine residues that are considered to be important for integrin binding [34].

### 2.2. Structure of the Rabbit ICAM-5 Gene and Protein

A full-length cDNA of rabbit reverse transcribed from rabbit hippocampus total ICAM-5 RNA is 3013 bp which includes an open reading frame of 2736 nucleotides beginning at position 125, terminates at nucleotide 2860 and two mRNA-destabilizing sequence motifs (ATTTA) in the 3′ untranslated region [4].

A hydrophobicity profile of the protein revealed that rabbit ICAM-5 has the typical features observed in type I integral membrane proteins. A potential initiating Met is followed by a putative signal sequence [35] with 29 amino acids. The overall identity of rabbit and mouse ICAM-5 is 78% at the nucleotide level and 82% at the amino acid level [4]. Rabbit ICAM-5 also contains nine tandem Ig-like domains in its extracellular region with intrachain disulfide bonds typical of Ig-like loops [36]. Domain I of ICAM-5 protein is unusual for an Ig-like domain: it contains 4 Cys residues with the potential to form two disulfide bridges. In contrast, domains II, III, and IV are highly homologous to the corresponding domains of ICAM-1 and -3/R. Domains V–VIII of ICAM-5 are highly conserved four tandem repeating units, each containing 81–93 amino acids. These domains are closely related to domain V of ICAM-1 and -3tR. All these domains contain 4 conserved Cys, 3 Pro, 3 Gly, 1 Trp, 1 Tyr, 1 Ala, and 1 Val. These Cys residues in domains V–VIII are arranged in a motif different from that found in domain I. Domain IX of ICAM-5 protein has all the conserved hallmarks of the C2-SET Ig-like domain [36]. Among the nine Ig-like domains of ICAM-5, only domain IX has a characteristic Trp residue between the 2 Cys residues. This type of domain is observed in a number of IgSF molecules expressed in the nervous system [37, 38]. The sequence of positions 30–821 is predominantly hydrophilic and is followed by a stretch of 30 hydrophobic residues, which is characteristic of a membrane-spanning domain. The cytoplasmic region at the C-terminus of the protein is 61 residues in length and this region has little homology with any ICAM members [4]. Instead, the middle portion (45 amino acids: residues 859–903) of the rabbit ICAM-5 cytoplasmic domain shows the highest homology with the N-terminal region of rabbit cardiac troponin-T2 [39]. Both sequences are highly acidic and contain a Gly-rich motif. Surprisingly, 10 out of the 11 residues (91%) are identical in these Gly-rich sequences of motif and troponin-T2. Among the known IgSF molecules, the cytoplasmic domain is most similar to the Ng-CAM cytoplasmic domain (1163–1218), with 39% identity [40]. In addition, the cytoplasmic domain of ICAM-5 contains one and two potential sites for phosphorylation by protein tyrosine kinases and by casein kinase II, respectively (Tyr-861, Ser-868, and Ser-884 in rabbit ICAM-5).The mature protein gives rise to a 883 amino acid polypeptide with an expected molecular weight of 92,762 and 14 potential Asn-linked glycosylation sites. Among other superfamily members, it is most similar to ICAMs 1, 2, and 3/R [24–27].

### 2.3. Structure of the Human ICAM-5 Gene and Protein

The human gene was mapped to the human chromosome 19p13.2, where the ICAM-1, -3, and -4 (LW) genes are located [5]. Genetically, four of the five human ICAMs (all but ICAM-2) are clustered on a region of chromosome 19p13.2 [5, 41, 42]. This clear indication of gene duplication events argues for the existence of a primordial ICAM, whose expression pattern and functional significance have expanded as new members have arisen. ICAM-5 has a long open reading frame of 2,772 nucleotides, a 59-untranslated region of 65 nucleotides, and a 39-untranslated region of 198 nucleotides, which contains a polyadenylation signal at 13 nucleotides upstream of the poly (A) tail in. Within the 39-untranslated region there are five ATTTA motifs, which are believed to confer instability to mRNA [43, 44]. There is one potential translation initiation site (ATG, nucleotides 66–68) surrounded by the nucleotides well matching with the Kozak's consensus sequence [45]. An in-frame termination codon TAG locates 54 nucleotides upstream of the initiator ATG. The human ICAM-5 gene is composed of 11 exons encoding the signal peptide, each of the nine extracellular domains, and with a single exon encoding both the transmembrane and the cytoplasmic domains [46]. Chen et al. [47] reported that there exist V301I and T348A polymorphisms in the gene. Allele frequencies differed in African Americans from published frequencies for Europeans. For instance, the minor allele frequencies in African Americans for the two ICAM-5 SNPs were both 9.2%, as compared to 34–44% among Germans and Australians. However, there is disagreement on this issue [48].

**Figure 1:**
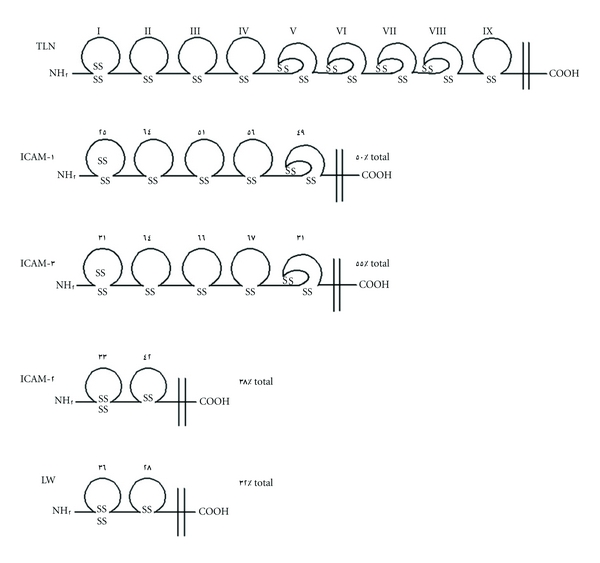
Structural comparison of with ICAM-1, -2, -3, and LW antigen (ICAM-4). *A*, amino acid sequence alignment of with ICAMs and LW antigen. The amino acid sequence of human (*domains I–V*) was aligned with those of ICAM-1 (*domains I–V*) (8, 9), ICAM-2 (*domains I *and *II*) (10), ICAM-3 (*domains I–V*) (11, 12), and LW antigen (*domains I *and *II*) (13). The *open circles *denote the highly conserved Cys residues. The residues conserved in more than three of the five members (*domains I *and *II*) or more than two of the three (*domains III–V*) are *highlighted*. *B*, proposed model of the domain structure of ICAMs and LW antigen. Amino acid identities between the corresponding Ig-like domains of ICAMs and LW antigen are shown with *Arabic numerals *above the Ig-like domains, respectively. Individual Ig-like domains are numbered *I–IX *on the top. Extra- and intracellular orientations are indicated by *NH_2_* and *COOH *on the protein chains, respectively. Intradomain disulfide bonds are represented by *SS* (Cited from [5]).

The human ICAM-5 is 84 and 85% identical with mouse and rabbit in amino acid sequence, respectively. The human ICAM-5 polypeptide contains two hydrophobic segments, which may serve as a signal peptide (27 residues at the NH_2_ terminus) and a transmembrane region (28 residues near the COOH terminus). Between the two hydrophobic regions, there is a large extracellular region (805 residues) with 15 potential N-linked glycosylation sites. A cytoplasmic region at the carboxyl terminus is 64 residues in length. The extracellular region of human ICAM-5 comprises nine tandemly arranged Ig-like domains, rendering it to be a member of the Ig superfamily [36, 37, 49]. In particular, the distal eight Ig-like domains are closely related to those of ICAMs [25, 30]. Total amino acid identity is 50% with ICAM-1 (domains I–V), 55% with ICAM-3 (domains I–V), 38% with ICAM-2 (domains I and II), and 32% with ICAM-4 (domains I and II). The highest homology is observed with the domain II of ICAM-1 and the domains II–IV of ICAM-3 with more than 64% amino acid identity (Figure 1). The presence of nine Ig-like domains and the position of all cysteine residues (28 in the extracellular region and 2 in the cytoplasmic region) are completely conserved in all the three species. Twelve N-linked glycosylation sites are conserved in human, mouse, and rabbit. 

## 3. Expression of ICAM-5

The expression of ICAM-5 protein is confined to the mammalian telencephalon of the central nervous system [3, 4, 50], whereas all the other ICAM members are expressed mostly by cells in the immune and blood systems, such as lymphocytes (ICAM-1 and -3), endothelial cells (ICAM-1 and -2), epithelial cells (ICAM-1), and erythrocytes (ICAM-4) [24, 25, 27, 29, 30]. Within the telencephalon, the expression of ICAM-5 is restricted to certain types of neurons. For example, in the adult rabbit olfactory bulb, granule cells, but not mitral and tufted cells express [51]; in the cat visual cortex, is absent in neurons within layer IV, which receives massive afferent input from the thalamus, but is present in neurons of other cortical layers [52]; in the rat hippocampus, expression is excluded from GABA- (*γ*-aminobutyric acid) energic inhibitory interneurons, but is expressed in nearly all excitatory pyramidal neurons [53]. Northern blot analysis demonstrated human mRNA of 3.0 kilobase pairs in the hippocampus; however, no signal was detected in the cerebellum [5]. 

At the subcellular level, ICAM-5 is localized exclusively on the membrane of filopodia as well as the membranes of cell bodies and dendrites but not on axons [6, 53, 54]. On cultured hippocampal neurons, ICAM-5 is expressed in dendritic growth cones and filopodia.

In rodents, ICAM-5 is almost absent from embryonic brain, appears around birth, increases dramatically during the postnatal weeks, and continues to be expressed at a high level in the adult brain [4]. Expression of ICAM-5 is developmentally regulated, with the first appearance around birth, a drastic increase in a few weeks postnatally, and persistence of high levels in adult life. Thus, the expression of ICAM-5 is brain segment-specific, neuron-specific, somadendritic membrane-specific, and develops mental stage-specific [3, 4, 50–52].

### 3.1. Expression of the Mouse ICAM-5 Gene and Protein

In situ hybridization was performed to determine in detail the spatial pattern of ICAM-5 expression. A parasagittal plane of adult mouse brain hybridized with antisense cRNA probe displaying that ICAM-5 mRNA was detected exclusively in telencephalic regions. No particular hybridizing signals were observed in a parallel control experiment with sense cRNA probe. It shows a boundary region between the telencephalon (caudate putamen) and the diencephalon (globus pallidus). Positive signals were seen only in neurons of the caudate putamen, and not in those of the globus pallidus. The telencephalon-restricted expression of ICAM-5 mRNA is consistent with the immunohistochemical results showing the expression of its protein. The mRNA was expressed strongly in granule cells of the dentate gyrus, pyramidal cells of the hippocampus, granule cells of the olfactory bulb, and neurons in layers II/111 and V/Vl of the cerebral neocortex. However, little or only weak signals were detected in several types of telencephalic neurons, such as mitral/tufted cells and periglomerular cells in the olfactory bulb and neurons in layer IV of the cerebral neocortex. These results indicate that within the telencephalon, ICAM-5 is expressed in a neuronal type-specific manner [4].

### 3.2. Expression of the Rabbit ICAM-5 Gene and Protein

The level of ICAM-5 mRNA in rabbit telencephalon is low at embryonic day 25, elevated dramatically by postnatal day 10, and remains high until adulthood [4, 7]. Immunohistochemistry has also revealed that the amount of ICAM-5 is small in the cerebrum of a newborn rabbit. At this stage, ICAM-5 is hardly detectable in most areas of the neocortex, and its localization is restricted to phylogenetically older regions such as the olfactory bulb, piriform cortex, corpus striatum, cingulate cortex, and hippocampus. After birth (postnatal day 0–10), the densities and areas of ICAM-5-positive structures rapidly increase [50]. Northern blot analysis with a full-length probe of rabbit ICAM-5 cDNA was done to survey the expression of mRNA during development, in adult brain regions and peripheral tissues showing that in embryonic day 25 rabbit telencephalon, a faint signal of transcript was detected at 3.2 kb; at postnatal day 10, the level of mRNA was dramatically increased, and the level remained high into adulthood. The expression of transcript was confined exclusively to telencephalic regions, including the olfactory bulb, olfactory cortex, cerebral neocortex, hippocampus, and striatum. No hybridizing signal was detected in other brain segments (diencephalon, mesencephalon, metencephalon, and myelencephalon) or in the spinal cord. In addition, transcript was not observed in peripheral tissues such as the heart, lung, kidney, spleen, intestine, and skeletal muscle [4].

### 3.3. Expression of the Human ICAM-5 Gene and Protein

In the adult human brain, immunoreactivity of ICAM-5 localized to the telencephalic gray matter: the cerebral cortex, hippocampus, and basal ganglia. The labeling intensity changed during development, and the time course varied among regions: the hippocampus showed a rapid increase in immunoreactivity of ICAM-5 in the late fetal period and temporal cortex in the early infancy, the cerebral white matter, as well as the other brain segments such as the thalamus, cerebellum, and brainstem, remained negative for at all the developmental stages [55]. In the cerebrum of a 25-gestational-week (GW) fetus, there was no immunoreactivity of ICAM-5. The hippocampus was the first structure to show positivity at 29 GW. At 29–33 GW, weak staining was noted in the neuropil of the cornu ammonis (molecular and pyramidal layers) and area dentata (molecular layer). At 35 GW, the overall immunoreactivity was comparable to that of an adult brain. In the molecular layer of the cornu ammonis, apical dendrites of the pyramidal neurons were intensely stained. Thereafter, the zone showing maximal staining moved from the middle to the upper third of the layer [55]. At first, labeling was weak and restricted to the cytoplasm of pyramidal neurons from 35 to 39 GW, but thereafter it became diffused and intense in the cortical layers. The staining was most intense in the molecular (1st) layer, moderate in the external (3rd), and internal pyramidal (5th) layers, and least intense in the external (2nd) and internal granular (4th) layers.

On the western blot of homogenates of the frontal cortex, ICAM-5 (130 kDa) was detectable in the tissues of three postnatal patients, whereas those of a 30-GW fetus and a 39-GW neonate contained no detectable amount of ICAM-5. Among the former, there was no significant difference in the content. In the neuropil of the temporal cortex, immunoreactivity of ICAM-5 appeared first at 2 postnatal months and was rapidly increased by 5 months. The blot thus demonstrated a sharp increase in the amount of ICAM-5 that occured during the early infancy [55]. In conclusion, the upregulation of ICAM-5 occurs in human cerebrum around birth, which is comparable to the previous results in animals and the suspected roles in constructing neuronal networks [4, 7, 51, 52].

To compare the development of ICAM-5 with that of two other markers of dendritic/synaptic development: MAP2 and synaptophysin. The former is a high molecular weight protein that facilitates intracellular transport, whereas the latter is a membrane glycoprotein of presynaptic vesicles. In the human cerebral cortex, MAP2 and synaptophysin are first detectable at 20 and 23 GW, respectively, according to previous developmental studies [56–58]. Immunoreactivities for synaptophysin and MAP2 appeared earlier than that for ICAM-5, being detectable in the 25 GW cerebrums. In the hippocampus and temporal cortex, the neuronal perikarya was positively stained for synaptophysin at 25 GW, and their dendrites at 32–36 GW. The peak level of expression was reached at 37–40 GW. MAP2 staining was noted in the neuronal perikarya and dendrites at 25 GW. The intensity was increased by 32–36 GW, and decreased thereafter [55]. These markers appear prior to 25 GW, when ICAM-5 is not yet detectable. Thus the development of ICAM-5 appeared to be associated with the late phase of dendritic/synaptic development. In conclusion, in human cerebrum, ICAM-5 appears in the hippocampus from the 29th gestational week, and in the temporal cortex from the 35th to 39th gestational weeks, intensifies during the perinatal period, and persists into adulthood. Thus, the developmental appearance of ICAM-5 parallels the time of dendritic elongation and branching, and synapse formation in the telencephalon.

## 4. Function of ICAM-5

### 4.1. Immunological Activities

Human ICAM-5 binds to the CD11a/CD18 integrin, and that the binding region in ICAM-5 is localized to the first five Ig domains [59]. ICAM-5 D1-Fc supported the binding of T cells at about 70% of the wild-type level, which was significantly higher than T cell binding to control human IgG [60]. The binding of T cells to ICAM-5 D1-D2-Fc was slightly higher than to ICAM-5 D1-Fc. The binding was almost completely abrogated when the first or the first two domains were deleted. The sixth domain of ICAM-5 supported T cell binding at about 60% of the wild-type level.

Protein constructs containing the first Ig domain of ICAM-5 were able to support CD11a/CD18 interaction, while deletion of the first domain abolished binding. Monoclonal antibodies reacting with the first domain of ICAM-5 also completely blocked the interaction. The soluble first domain of ICAM-5 inhibited the binding of T cells to immobilized ICAM-5 at concentrations of 50 nM and higher. Interestingly, the sixth domain of ICAM-5 was also able to support leukocyte binding, but this binding activity may not involve leukocyte integrins. To test the involvement of ICAM-5 in leukocyte-neuron interactions, an assay using human T cells binding to rat hippocampal neurons was established. This binding was blocked by monoclonal antibodies against CD11a/CD18 and ICAM-5 [60]. Thus ICAM-5 may act as a major adhesion molecule for leukocyte binding to neurons in the central nervous system.

ICAMs mediate intercellular adhesion events also through interaction with a common counter receptor leukocyte function-associated antigen-1 (LFA-1) integrin in the nervous and immune systems, respectively [5, 34, 59]. Thus, LFA-1 integrin has an intimate relationship with ICAMs both structurally and functionally. ICAM-5 as a candidate for postsynaptic elements to contact leukocyte function-associated antigen-1 (LFA-1), a major leukocyte integrin expressed in lymphocytes and microglia, was precisely opposed to CD18-positive structures in presynaptic elemente [61]. ICAM-5 has been shown to bind to LFA-1 [5, 59, 60] and to regulate the morphology of microglia [62]. In the central nervous system, LFA-1 is selectively and constitutively expressed by microglia, suggesting that ICAM-5-LFA-1 binding may mediate cell-cell interactions between telencephalic neurons and microglia. ICAM-5 induced an intensive spreading of lamellipodia, causing a rapid change in microglial morphology. In contrast, ICAM-5 induced no significant change in morphology of neuroblastoma and fibroblasts. Furthermore, the ICAM-5-induced spreading of microglia was accompanied by a clustering of LFA-1 on cell surface membrane [62]. These results provide evidence that ICAM-5 binding to the surface of microglia transduces signals into microglia that mediate or accelerate cell spreading and LFA-1 redistribution, implying that neuronal ICAM-5 may control the state and function of microglia in both physiological and pathological conditions. Data also suggest that ICAM-5 plays a critical role in modulating chemokine production in the CNS [63].

In conditions of brain ischemia [64], epilepsy [65, 66], and encephalitis [67], the soluble form of ICAM-5/sICAM-5 has been detected in physiologic fluids. Tian et al. [68] find that the expression of ICAM-5 is not upregulated by the cytokines tumor necrosis factor (TNF) and interferon-*γ* (IFN-*γ*). Activated T cells promote the cleavage of ICAM-5 from neurons, which results in the formation of the sICAM-5. Whereas sICAM-1 acts as a costimulatory, sICAM-5 suppresses the T-cell receptor- (TCR-) mediated activation of T cells as indicated by the decreased expression of CD69, CD40L, and CD25 (IL-2R) on T cells, especially CD45RO^Low^ naive T cells. Moreover, sICAM-5 promotes the expression of transforming growth factor-*β*1 (TGF-*β*1) and IFN-*γ*, but not TNF [68]. SICAM-5 attenuates the T-cell receptor-mediated activation of T cells as demonstrated by the decreased expression of the activation markers CD69, CD40L, and CD25 (IL-2R). This effect is most clearly seen in CD45RO^Low^ (naive), and not in CD45RO^High^ (memory) T cells, and is most effective early in priming, but not in the presence of strong costimulatory signals. Furthermore, sICAM-5 promotes the mRNA expression of the cytokines TGF-*β*1 and IFN-*γ*, but not TNF. ICAM-5 can bind to LFA-1 that is expressed on the surface membrane of microglia. It is also possible that the LFA-1-ICAM-5 binding might mediate the signal for the activation of microglia. Actually, ICAM-5 is not involved in the pathological changes in the cell-to-cell contacts between microglia and pyramidal cell dendrites [69]. Study of CSF samples of patients with acute encephalitis and MS showed that sICAM-5 was increased in encephalitis (320 ± 107 ng/mL; *n* = 25), as compared with patients with MS (128 ± 10 ng/mL; *n* = 16) and control subjects without CNS disease (137 ± 6 ng/mL; *n* = 42) [67]. It suggests that sICAM-5 is cleaved from CNS into CSF during acute encephalitis, and it may mediate leukocyte-neuron interactions. SICAM-5 release from cerebral neurons may actively regulate immune responses and leukocyte adhesion during microbial neuroinvasion in humans during encephalitis. Importantly, ICAM-5 can be cleaved under pathological conditions and released into the cerebral-spinal fluid and blood. Soluble ICAM-5 was detected in experimental hypoxic-ischemic injury of the brain [64] and encephalitis [67]. The ICAM-5 immunoassay may turn out to be clinically useful in the differential diagnosis of brain diseases [67]. In fact, it attenuates T cell activation, evidently through its influence on the expression of various cell surface receptors and cytokines [68]. Moreover, ICAM-5-mediated neuronal regulation of T cell activation is aimed at a very specific early transitional stage, which provides a relevant mechanism in acute versus chronic neuroinflammation. Thus, ICAM-5 may interfere with the formation and function of immunological synapses, and strengthen the immune privilege of the brain. This function may turn out to be pivotal in the brain, where inflammation often is deleterious.

### 4.2. Neuritis Outgrowth-Promoting Activity

During development, ICAM-5 appears in parallel with dendritic elongation and branching, as well as synaptic formation in the telencephalon [4, 51, 52]. ICAM-5 immunoreactivity appeared at 29 gestational weeks (GW), intensified subsequently, and persisted into adulthood. In the temporal cortex labeling was weak and restricted to the cytoplasm of pyramidal neurons from 35 to 39 GW, but thereafter became diffuse and intense in the cortical layers, especially the molecular layer, by 5 months of postnatal age. The development of ICAM-5 was late compared to synaptophysin and microtubule-associated protein 2, suggesting its involvement in the functional maturation of neuronal dendrites and synapses [55]. In agreement, ICAM-5 has been shown to promote dendritic elongation and branching of hippocampal neurons in vitro [70]. Surface coated ICAM-5-Fc promoted dendritic outgrowth and arborization of ICAM-5-expressing hippocampal neurons [70].

ICAM-5-deficient mice exhibited decreased density of dendritic filopodia and accelerated maturation of dendritic spines [71]. ICAM-5 facilitates the formation and maintenance of dendritic filopodia and thereby slows spine maturation in hippocampal neurons. Tamada et al. [72] examined neurite outgrowth-promoting activity of ICAM-5 by using a culture of hippocampal cells dissociated from E16 mouse. After 24 h in culture, many neurons extended neurites on the plates coated with recombinant ICAM-5/Fc chimeric protein which was composed of the extracellular region of mouse ICAM-5 and the Fc region of human IgG1. In contrast, only a few neurons extended neurites on the control plates without ICAM-5/Fc. The length of neurite showed nonlinear dose-dependent relationship with the concentration of ICAM-5/Fc. Since human IgG did not show any significant neurite outgrowth-promoting activity, the neurite outgrowth on ICAM-5/Fc appears to be caused by the extracellular region of ICAM-5, but not by the Fc region of IgG. Although ICAM-1 has a structure closely related to ICAM-5, only a negligible neurite outgrowth was found on ICAM-1/Fc even at the highest concentration (100 mg/mL). Neurite outgrowth was greatly reduced when ICAM-5/Fc substrates were blocked by the incubation with the anti-ICAM-5 antibody. On the other hand, neurite outgrowth on laminin substrates was not significantly changed by the incubation with the same antibody. These results confirm that ICAM-5 has a neurite outgrowth-promoting activity.

In vitro studies also show that ICAM-5 colocalized with both *α*-actinin and F-actin and ICAM-5-cytoskeletal association is involved in neuritic outgrowth [73]. It found that the ICAM-5/*α*-actinin interaction is involved in neuritic outgrowth and the ICAM-5_ 857–861_ cytoplasmic peptide induced morphological changes in Paju-ICAM-5 cells. It indicates that the interaction between ICAM-5 and *α*-actinin is mediated through binding of positively charged amino acids near the transmembrane domain of ICAM-5, and this interaction may play an important role in neuronal differentiation. ICAM-5 is mainly located in dendritic filopodia and immature thin spines. Soluble ICAM-5 promotes elongation of dendritic filopodia from wild-type neurons, but not from ICAM-5-deficient neurons. ICAM-5 deficiency also causes retraction of thin spine heads in response to NMDA stimulation [74].

The mechanism of ICAM-5 neurite outgrowth-promoting activity is that ICAM-5 has a homophilic binding activity [70] and ICAM-5-ERM interaction [75]. On the one hand, the homophilic adhesion of ICAM-5 mediates induction of dendritic outgrowth [72]. On the other hand, ICAM-5 cytoplasmic region binds ERM (ezrin/radixin/moesin) family proteins that link membrane proteins to actin cytoskeleton. Besides, ICAM-5 ectodomain can interact with *β*(1) integrins, and it can stimulate *β*(1) integrin-dependent phosphorylation of cofilin, an event that has previously been linked to MMP-dependent spine maturation [76]. The two things may lead to activation of signal transduction.

### 4.3. Dendrites' Targeting Signals

A full-length ICAM-5 ectopically expressed in the Purkinje cells was localized exclusively to dendrites but not to axons. A deletion of cytoplasmic C-terminal 12 amino acids (residues 901–912) or a point mutation of Phe905 to Ala abrogated the dendrite specific targeting with appearance of the truncated and point-mutated ICAM-5 in both axons and dendrites. The cytoplasmic region of ICAM-5 consists of ~60 amino acids that are highly conserved across animal species. Furthermore, an addition of the C-terminal 17 amino acids (residues 896–912) of ICAM-5 to an unrelated molecule (CD8) was sufficient for its specific targeting to dendrites in several types of neurons [54]. This result suggests that an open structure of the ICAM-5 C terminus is important to execute selective targeting into dendrites. Because the C-terminal region of ICAM-5 does not contain any canonical dendritic targeting sequences such as the tyrosine-based motif or the dileucine motif, this study suggests a novel mechanism of protein trafficking to the dendritic compartment of neurons. The cytoplasmic region of ICAM-5 contains only one Tyr at 861 and one Phe at 905. The ICAM-5-Y861A mutant showed dendrite-specific localization in Purkinje cells, similar to the full-length ICAM-5. In contrast, the ICAM-5-F905A mutant was detected both in the dendrites and axons of Purkinje cells, exhibiting no preference of localization. These results suggest that Phe905, but not Tyr861, is important for the dendrite-specific targeting of ICAM-5. Although ICAM-5 cytoplasmic region is sufficient for dendritic targeting in Purkinje cells, ICAM-5 dendritic targeting signal does not serve as basolateral targeting signal in polarized epithelial cells [54]. The important role of ICAM-5-ERM interaction in the formation of dendritic filopodia, which leads to subsequent synaptogenesis and establishment of functional neural circuitry in the developing brain, also shows that proteins integrate multiple signals at the cell cortex. Members of the ERM family link integral plasma-membrane proteins with underlying actin cytoskeleton, participate in signal transduction pathways, and mediate the formation of specialized membrane protrusions such as microvilli, uropods, and ruffles [77, 78]. In cultured hippocampal neurons, phosphorylated active forms of ERM proteins are co-localized with ICAM-5 in dendritic filopodia, whereas *α*-actinin, another binding partner of ICAM-5, is co-localized with ICAM-5 at surface membranes of soma and dendritic shafts. Expression of ICAM-5 constitutively active ezrin induces dendritic filopodia formation, whereas small interference RNA-mediated knockdown of ERM proteins decreases filopodia density and accelerates spine maturation [75]. The results indicate the important role of ICAM-5-ERM interaction in the formation of dendritic filopodia, which leads to signal transduction and subsequent synaptogenesis and establishment of functional neural circuitry in the developing brain.

### 4.4. Association between ICAM-5 and Cognition

In 1998, Rieckmann et al. confirmed that ICAM-5 as an indicator for temporal-lobe dysfunction [65]. They found that significantly higher serum concentrations of sICAM-5 in patients with acute herpes-simplex encephalitis (122 ng/mL) and established diagnosis of intractable temporal lobe epilepsy (48 ng/mL) than in healthy donor (0.8 ng/mL) or patients with other neurological disease. It is hypothesized that altered local function of the frontotemporal cortex in localization-related epilepsy might be better predicted by the biochemical marker ICAM-5 than epilepsy characteristics such as seizure focus because there is an association between decreased frontotemporal activation on fMRI, both detectable ICAM-5 serum levels and levetiracetam use [79]. However, there is no significant differences observed between ICAM-5 serum levels in febrile seizures and controls in children [66].

ICAM-5 immunoreactivity was markedly decreased in the brain of Alzheimer's disease (AD) patients, particularly in the hippocampal formation [80]. In AD brain, the intensity of neuropil labeling for ICAM-5 was decreased in the neocortex and more sharply in the hippocampal formation. In hippocampal CA areas where the neuropil labeling was completely depleted, a small number of neurons and neuronal processes, particularly the apical dendrite of pyramidal neurons were stained rather intensely. Nissl staining of nearby sections indicated that such ICAM-5 positive neurons represented only a few of the remaining neurons in the area. In a small number of cases, a few neurofibrillary tangles were stained weakly for ICAM-5. Double immunostaining for ICAM-5 and C4d revealed that C4d-positive ghost tangles were negative for ICAM-5. The hippocampal structure is considered to be involved in memory function in which synaptic plasticity plays an important role. Disruption of cell-cell interactions, which might be normally maintained by ICAM-5, would cause functional impairment of the neuronal networks. The loss of ICAM-5 in the hippocampus thus might be relevant to dementia in AD patients. It has yet to be determined whether the reduction of ICAM-5 expression precedes or parallels the neuronal degeneration. In some ICAM-5 depleted areas a few of the remaining neurons and their dendrites were stained rather intensely. This might indicate upregulation of ICAM-5 expression by some surviving neurons, although the reduced neuropil labeling could also account for the visualization of these neurons.

Blocking of ICAM-5 function using anti-ICAM-5 antibody or recombinant soluble ICAM-5 protein caused a striking suppression of the long-term potentiation (LTP) at the Schaffer collateral-CA1 synapses. The suppression was observed even when the blocking was initiated immediately after the tetanic stimuli. These observations suggest a role for ICAM-5-mediated cell-cell interactions as a key step in the development of LTP [81].

Independent in vitro and in vivo compelling evidence showed that ICAM-5 binds to Presenilin 1 and 2 (PS1 and PS2). Importantly, the interaction was also established at the endogenous level of expression. It demonstrated that the interaction is functional and not structural [82]. PS1 deficiency results in the abnormal accumulation of ICAM-5 in intracellular compartment. The first transmembrane domain and carboxyl terminus of PS1 form a binding pocket with the transmembrane domain of ICAM-5. Remarkably, APP binds to the same regions via part of its transmembrane domain encompassing the critical residues mutated in familial AD. It indicates a spatial dissociation between the binding site and the proposed catalytic site near the critical aspartates in PSs. ICAM-5 binds to the membrane spanning region in presenilins as does APP, and the transmembrane region of presenilins is often mutated in AD patients [82]. However, ICAM-5 is not a substrate for *γ*-secretase cleavage; it displays a prolonged half-life in PS1^−/−^ hippocampal neurons [83]. ICAM-5 may also be important in the development of AD as evidenced by its association with presenilins and APP.

## 5. Conclusion

ICAM-5 is a cell surface glycoprotein whose expression is confined exclusively within the mammal's telencephalon [3, 7, 50]. With its nine extracellular Ig domains, ICAM-5 is the largest member of ICAM subgroup identified so far. Therefore it is much more complex than the other ICAMs. The expression of ICAM-5 is confined to the telencephalic neurons of the central nervous system, whereas all the other ICAM members are expressed mostly by cells in the immune and blood systems. The developmental appearance of ICAM-5 parallels the time of dendritic elongation and branching, and synapse formation in the telencephalon.

ICAM-5 is expressed by subsets of the telencephalic neurons, but not by glial cells. In the neurons, ICAM-5 is localized to somadendritic membrane, but not to axonal membrane. In the course of brain development, ICAM-5 first appears around birth when the dendritic outgrowth and branching, spine formation, and synapse formation occur in the telencephalic regions. Afterwards, ICAM-5 expression persists even in adulthood. These unique expression patterns of ICAM-5 in brain segment-, neuronal subsets-, somadendritic membrane-, and developmental stage-specific fashions suggest that ICAM-5 may be a crucial cell surface molecule for the formation, maintenance, and plasticity of neuronal networks in the brain [3, 7, 50–52]. Since ICAM-5 is likely to be involved in the formation and maintenance of neuronal circuits in the telencephalon [4], it would be interesting to examine how ICAM-5 expression is altered in various human diseases that affect the intellectual and motor functions. ICAM-5 is a fascinating molecule. On the one hand it binds to leukocytes through the integrin and can inactivate immune cells. Most interestingly, ICAM-5 may also suppress immune functions by inhibition of T cell activation. This inhibitory activity could be important for limiting inflammatory damage in the brain. On the other hand, the main physiological functions of ICAM-5 are probably in the development and maturation of neuronal synapses.

## Abbreviations

AxCAM: Axon-associated cell adhesion molecule
CD: Cluster of differentiation
NCAM: Neural cell adhesion molecule
DenCAM: Dendrite-associated cell adhesion molecule
ECM: Extracellular matrix
ICAM: Intercellular adhesion molecule
Ig: Immunoglobulin
IgSF: Immunoglobulin superfamily
kDa: Kilodalton
LFA: Leukocyte function-associated antigen
LTP: Long-term potentiation
mAb: Monoclonal antibody
ICAM-5: Intercellular adhesion molecule-5.
